# Eagle syndrome causing cerebral sinus hypertension: Case report

**DOI:** 10.1016/j.radcr.2023.05.023

**Published:** 2023-06-03

**Authors:** Erik Werheim, Zachary Sokol, David Brown, Martin Oselkin

**Affiliations:** a Department of Neurosurgery, St Luke's University Health Network, 801 Ostrum St, Bethlehem, PA 18015, USA; b Department of Neurosurgery, Lewis Katz School of Medicine at Temple University, Philadelphia, PA; c St Luke's University Health Network, Bethlehem, PA

**Keywords:** Eagle Syndrome, Cerebral sinus hypertension, Internal jugular stenosis, Styloidectomy, Dynamic cervicocerebral angiography, Dynamic cervicocerebral venography

## Abstract

Eagle Syndrome is a rare condition with a variety of presentations, resulting from an enlarged styloid process or calcified stylohyoid ligament. Due to the variety of presentations, diagnosis can be difficult. In this report, we present a case of ES that presented with a constellation of neurological symptoms, including headache and visual disturbance, ultimately found to be due to cerebral sinus hypertension, exacerbated by certain movements, caused by an enlarged styloid process with calcification of the stylohyoid ligament, consistent with ES. The patient underwent styloidectomy with immediate resolution of symptoms. This case report illustrates the diagnostic quandary often posed by ES and hopes to add further understanding to its presentation and diagnosis.

## Introduction

Eagle Syndrome (ES) is a rare condition that results from an enlarged styloid process or calcified stylohyoid ligament. Initially described by Eagle in 1937, the basic pathophysiology involves compression or stretching of nerve and vascular components in the retrostyloid compartment [1]. Typical symptoms include craniofacial and pharyngeal pain, however studies have shown ES can elicit a plethora of nonspecific symptoms and varying clinical presentations, making it difficult to diagnose and treat properly [2], [3], [4]. Conservative therapy consisting of analgesics, anticonvulsants, antidepressants, and/or local steroid injections can aid in management of mild cases of ES [3], [4], [5], [6]. Severe and refractory presentations warrant surgical intervention in the form of a styloidectomy done by either an intraoral (transpharyngeal) approach or an extraoral (cervical) approach [7]. Here we report a case of ES in a 71-year-old woman presenting with a chronic history of unexplained neurological symptoms.

## Case presentation

**Fig. 1:**
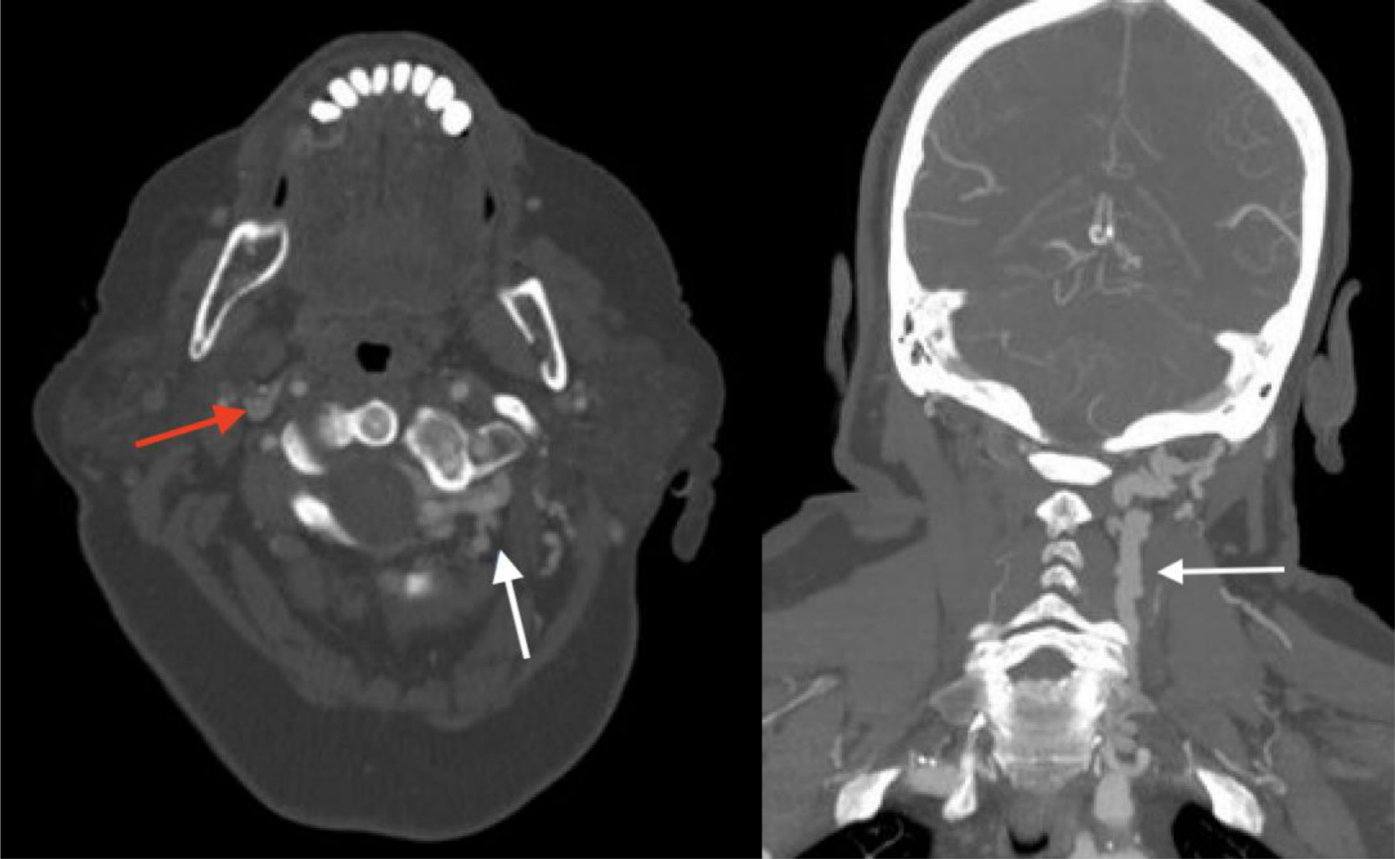
CTA axial (left), coronal (right): Prominence of left paraspinal veins (white arrow). Asymmetric and prominent vertebral venous drainage on the left initially concerning for an underlying vascular malformation. Red arrow demonstrates R internal jugular vein (IJV).

A 71-year-old woman initially presented with headaches and left retro-orbital pain. She had a long history of left sided headaches, left eye blurry vision, left ear and neck pain, along with neck swelling and a “thumping” sensation like her heartbeat. She received cervical epidural steroid injections, however she continued to have severe quality of life depreciation. Raising of the left arm in addition to certain head or neck positions exacerbated her symptoms. Of note, she had sustained a left neck trauma from a self-described “karate chop” when she was a child. Her neurologist ordered a CT angiogram to exclude a vascular explanation for her symptoms such as dissection. This ultimately revealed prominent venous structures in the left paraspinal musculature extending from the skull base to the internal jugular vein prompting concern for an underlying arteriovenous fistula (Fig. 1).

Red arrow demonstrating decreased enhancement, ultimately found to be stenosis due to ES.

**Fig. 2:**
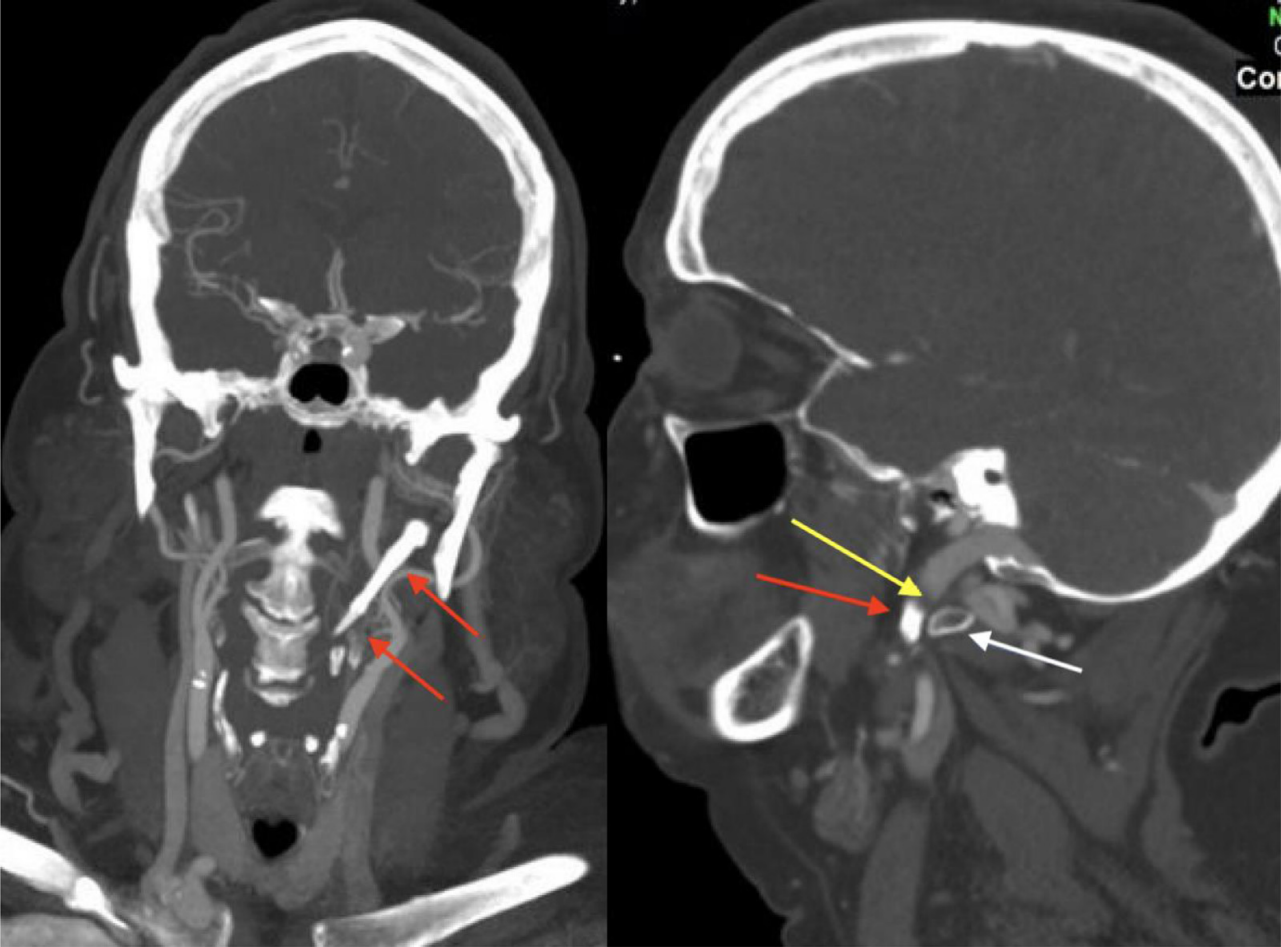
(A) (left) CTA coronal section, (B) (right), CTA sagittal section: demonstrating a markedly calcified and enlarged stylohoid ligament (yellow arrows) seen in the left neck and abuts the transverse process of C1 (white arrow). The left internal jugular vein (yellow arrow) is severely compressed by these two structures impeding flow.

**Fig. 3:**
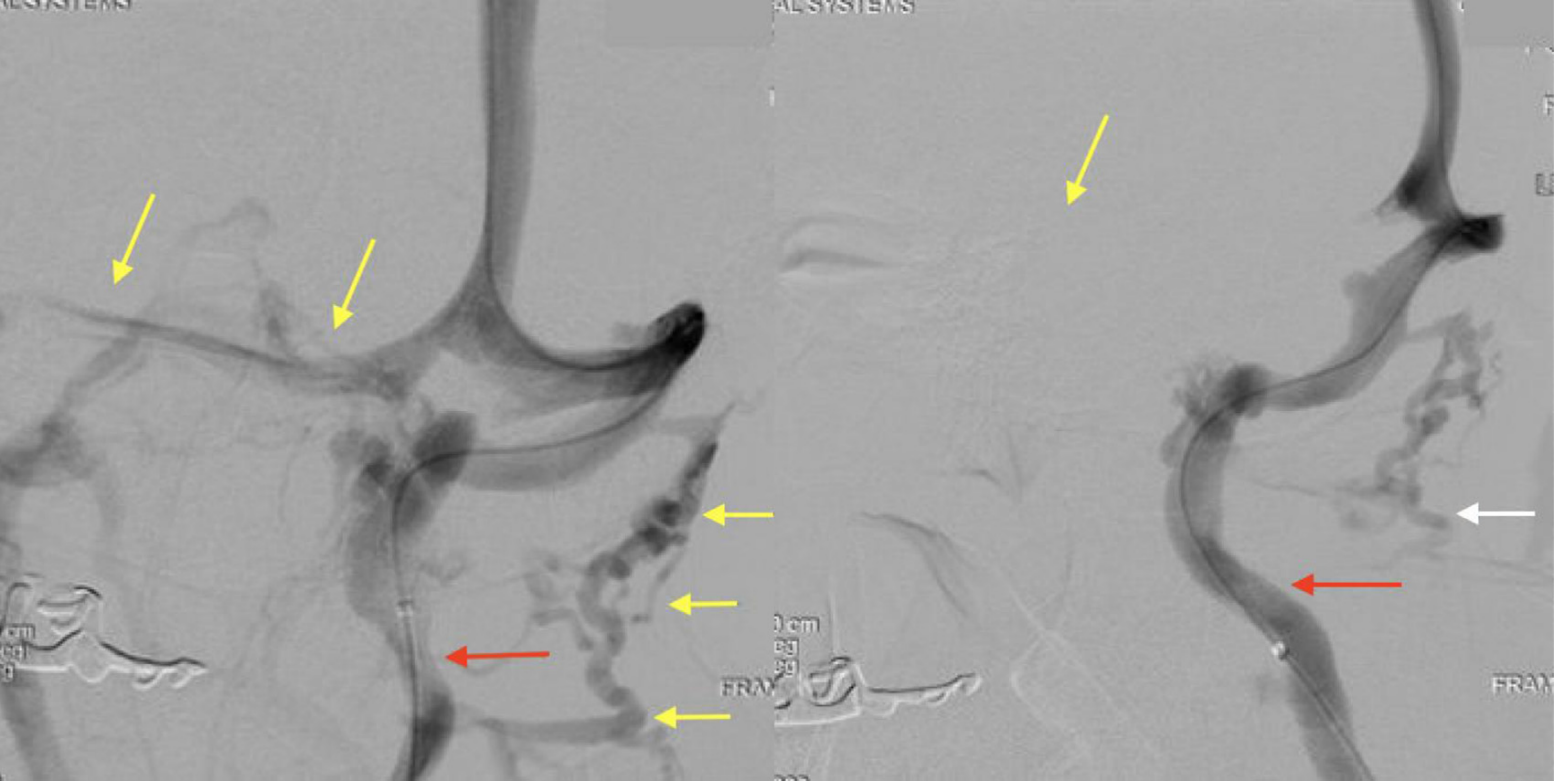
(A) (right):Cerebral venogram with catheter tip in the superior sagittal sinus and the head turned to the right. There is relative stenosis of the proximal left internal jugular (IJ) vein (red arrow). There is drainage into the relatively hypoplastic right transverse sinus as well as paraspinal collaterals on the left (white arrow). (B) (left) Venogram with the head in the neutral position shows a widely patent left IJ vein (red arrow). Also noticeably absent is drainage into the right transverse sinus (yellow arrow in expected location) and fewer paraspinal veins (white arrow).

Neurosurgical and ENT consultations were obtained and ultimately the paraspinal vein prominence was felt to be due to compression of the left internal jugular vein (IJV) (Fig. 2). To determine if this was a symptomatic lesion with intracranial pressure (ICP) change, dynamic cervicocerebral angiography and venography was performed with simultaneous venous manometry. This demonstrated proximal left IJV stenosis with head turning to the right (Fig. 3A) and relieved with turning to the neutral position (Fig. 2B).  Manometry with a right head turn revealed pressures in the left sigmoid sinus and left IJ proximal to the stenosis was approximately 10 mm Hg higher than when the same measurements were performed with the head in the neutral position while the pressure distal to the stenosis remained the same in all positions. Dynamic cerebral venography with manometry further confirmed the CTA findings of left IJ compression and illustrated changes in ICP with head movement to the right.

The pathophysiology responsible for her symptoms involved both a prominent, calcified stylohyoid ligament and an enlarged styloid process. These two anatomical variations lead to impingement on her left internal jugular against the transverse process of the cervical spine. A diagnosis of ES was made, and she was subsequently treated with a left sided styloidectomy. A transcervical styloidectomy was performed using a standard parotid approach and intraoperative facial nerve monitoring. Care was taken when skeletonizing the internal jugular vein as it was near the elongated styloid process. The styloid was divided proximally to its intersection with the internal jugular. Further removal of periosteum allowed for removal of an additional centimeter of thickened styloid. A drain was placed, and no neurological defects were found when the facial nerve was stimulated.

**Fig. 4:**
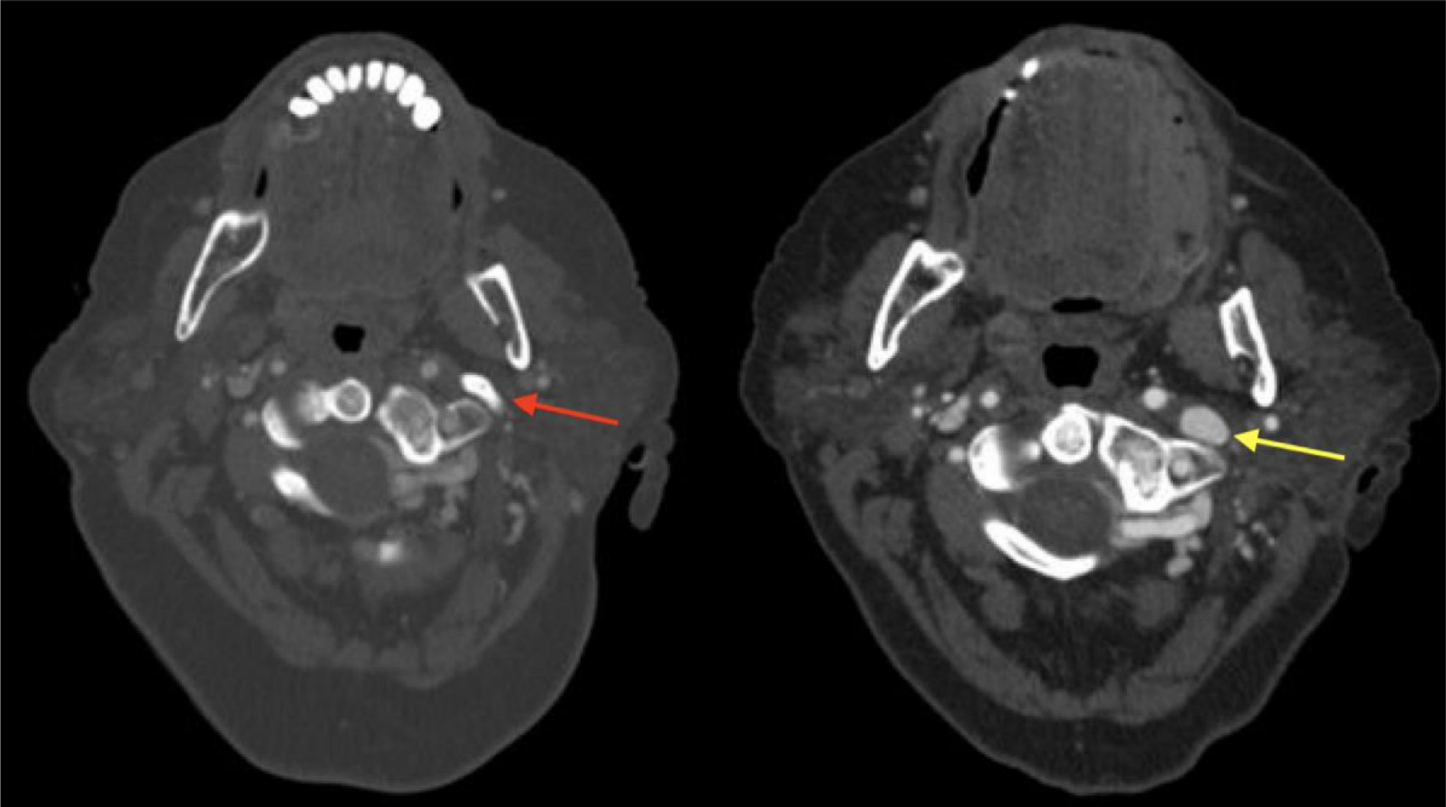
(A) (right): Preoperative CTA demonstrates calcified stylohyoid ligament with absence of the left internal jugular vein. (B) (left): yellow arrow demonstrating decompression of L IJV after styloidectomy.

The patient experienced a resolution of headache and pain symptoms post-operatively and was discharged the following day. She had mild facial nerve weakness and first bite syndrome which gradually resolved over the course of the following months. Postoperative imaging revealed resection of the lesion and restored patency of the left IJV (Fig. 4). Clinically, she had no residual whooshing or pulsatile tinnitus as well as resolution of her facial pain and headaches.

## Discussion

The retrostyloid compartment contains a variety of important structures ranging from the internal carotid artery, internal jugular vein, sympathetic nerve fibers, and cranial nerves IX-XII. The abnormal anatomy described in ES, like an elongated styloid process or a calcified stylohyoid ligament, can compress, occlude, and irritate these structures leading to a myriad of presenting symptoms [2], [3], [4]. The “classic” subtype of ES typically presents postpharyngeal trauma or surgery with common symptoms such as ear, jaw, or neck pain, dysphagia, and tinnitus [2], [3], [4]. Some of these symptoms were present in our case, however our patient's only significant trauma occurred when she was a child and although her symptoms were long standing, it may be hard to trace them back to an inciting event. There have been theories proposing anatomical variants revolving around endocrine dysfunction or rheumatoid degeneration leading to ossification/calcification of the stylohyoid ligament and styloid process [3]. This may have been a potential etiology of ES in this case, but it is difficult to say without further testing nor would it have necessarily guided the treatment approach chosen.

The second subtype of ES coined the “stylocarotid” syndrome occurs when an elongated styloid process impinges upon the internal carotid and is correlated to more severe sequelae [2], [3], [4]. There have been reports of presentations ranging from isolated Horner syndrome to carotid artery dissection and stroke [8], [9], [10]. Malik et al. [11] reported a case of glossopharyngeal neuralgia secondary to an elongated styloid process leading to syncope and atonic seizures. The pathophysiology in this case does not fully fit into either of the 2 subtypes as her symptoms involve the jugular nor did they present after an acute trauma. Although her symptoms were not acutely life threatening, she had a significant loss in quality of life warranting treatment.

Conservative treatment for ES can range from topical heat to epidural steroid injections with NSAIDs, analgesics, anticonvulsants, and anti-depressants falling somewhere in-between When conservative management fails, surgical treatment is an option and is typically more definitive, with longer standing symptom relief [[2], [3], [4],^7^,^12^]. There are 2 traditional approaches for the surgical treatment of ES, with a common goal of shortening an elongated styloid process. The procedure can be done in an intraoral (transpharyngeal) or extraoral (transcervical) fashion, our case details the latter. The intraoral approach is simpler, has a shorter operation time, and avoids potential cosmetic facial scarring. However, visualization of the styloid process is limited and there is a chance of postoperative airway edema [[2], [3], [4],^7^]. Regarding the transcervical approach, a clinical pearl is that greater exposure to the styloid process and its adjacent structures allows a more proximal resection and better visualization of important vascular and nervous anatomy. A greater concern in the transcervical approach is damage to the mandibular branch of the facial nerve [2], [3], [4]. Proper technique, visualization, and facial nerve monitoring can help avoid this complication as illustrated in this case. Ultimately, surgeon preference dictates approach as both can yield successful long-term outcomes.

## Conclusion

ES is scarcely seen, and it cohabits a broad differential for causes of headache and chronic craniofacial pain. Diagnosing ES can be difficult, but treatment offers favorable outcomes and a good long-term prognosis. Mild cases can be managed with analgesics and anti-inflammatory medication, whereas persistent and severe presentations can benefit from surgical intervention. We suggest including ES in the differential diagnosis for unexplained neurological symptoms. Correctly diagnosing and treating ES can provide substantial improvement in quality-of-life measures and avoid overtreatment without favorable results. This case report depicts the efficacy of styloidectomy in the treatment of ES.

## Patient consent

Written informed consent was obtained from the patient.
